# Preparation, Characterization, and Antioxidant Properties of Self-Assembled Nanomicelles of Curcumin-Loaded Amphiphilic Modified Chitosan

**DOI:** 10.3390/molecules29112693

**Published:** 2024-06-06

**Authors:** Qizhou Chen, Yuwei Jiang, Linlan Yuan, Lifen Liu, Xufeng Zhu, Rimeng Chen, Zhuo Wang, Kefeng Wu, Hui Luo, Qianqian Ouyang

**Affiliations:** 1 School of Ocean and Tropical Medicine, Research Center of Nano Technology and Application Engineering, Guangdong Medical University, Zhanjiang 524023, China; sj13423409060@gmail.com (Q.C.); yll1394178376@163.com (L.Y.); xufeng910730@126.com (X.Z.); winokhere@sina.com (K.W.); luohui@gdmu.edu.cn (H.L.); 2 Zhanjiang Institute for Drug Control, Zhanjiang 524023, China; 3 Guangdong Provincial Key Laboratory of Aquatic Product Processing and Safety, College of Food Science and Technology, Guangdong Ocean University, Zhanjiang 524023, China

**Keywords:** amphiphilic chitosan, curcumin, nanomicelles

## Abstract

Curcumin (Cur) is a phytochemical with various beneficial properties, including antioxidant, anti-inflammatory, and anticancer activities. However, its hydrophobicity, poor bioavailability, and stability limit its application in many biological approaches. In this study, a novel amphiphilic chitosan wall material was synthesized. The process was carried out via grafting chitosan with succinic anhydride (SA) as a hydrophilic group and deoxycholic acid (DA) as a hydrophobic group; ^1^H-NMR, FTIR, and XRD were employed to characterize the amphiphilic chitosan (CS—SA—DA). Using a low-cost, inorganic solvent-based procedure, CS—SA—DA was self-assembled to load Cur nanomicelles. This amphiphilic polymer formed self-assembled micelles with a core–shell structure and a critical micelle concentration (CMC) of 0.093 mg·mL^−1^. Cur-loaded nanomicelles were prepared by self-assembly and characterized by the Nano Particle Size Potential Analyzer and transmission electron microscopy (TEM). The mean particle size of the spherical Cur-loaded micelles was 770 nm. The drug entrapment efficiency and loading capacities were up to 80.80 ± 0.99% and 19.02 ± 0.46%, respectively. The in vitro release profiles of curcumin from micelles showed a constant release of the active drug molecule. Cytotoxicity studies and toxicity tests for zebrafish exhibited the comparable efficacy and safety of this delivery system. Moreover, the results showed that the entrapment of curcumin in micelles improves its stability, antioxidant, and anti-inflammatory activity.

## 1. Introduction

Curcumin is a lipophilic polyphenol derived from the rhizome of *Curcuma longa*, a member of the ginger family. It has been used for medicinal purposes for centuries in China and India [1]. Modern pharmacological studies have confirmed that curcumin has various biological effects, including anti-inflammatory, antioxidant, antibacterial, antiviral, and antitumor activities [2,3]. Thus, it has a wide range of applications. However, the shortcomings of curcumin, such as easy decomposition when exposed to light, easy oxidation, poor water solubility, and low bioavailability, limit its therapeutic and clinical applications [4,5,6]. Wahlstrom et al. [7] first reported that nearly 75% of curcumin was excreted via the feces and urine following oral administration in Sprague-Dawley rats. Usually, to increase the efficacy of a drug, it is necessary to increase its concentration in the blood. Changing the dosage form effectively increases the blood concentration of insoluble drugs, which is a hot research topic. In recent years, curcumin has been prepared as liposomes [8], polymeric micelles [9], solid dispersions [10], microcapsules [11], microspheres [12], microemulsions [13], and cyclodextrin inclusion compounds [14]. MelanieKolter et al. [15] prepared curcumin liposomes by hydrophobic thin-film and hand-extrusion methods. It was found that curcumin liposomes showed excellent physicochemical properties and storage stability. In general, the new dosage forms of curcumin have somewhat improved the low water solubility and bioavailability of curcumin. However, the new dosage forms still have the disadvantages of low drug loading capacity, large drug delivery volume, many excipients, and toxicity.

Nanomicelles are a new class of drug carriers consisting of block or graft copolymers with both hydrophilic and hydrophobic groups. The latter two can self-assemble in an aqueous solution to form nanocarriers with a core–shell structure. Nanomicelles have excellent drug delivery functions and are commonly used to deliver hydrophobic drugs [16]. Nanomicelles have received significant attention in nanomedicine because of their simple preparation, low cost, significantly improved drug solubility, increased drug bioavailability, and reduced toxic side effects [17,18,19]. For example, curcumin nanomicelles [20] were prepared and investigated using 1,2-distearoyl-sn-glycerol-3-phosphoethanolamine-N-methoxy-poly (ethylene glycol 2000) (DSPE-PEG) by the thin-film rehydration/reconstitution method. The water solubility and stability of curcumin were found to be significantly improved, especially its ability to enhance the therapeutic effect of curcumin in the treatment of cisplatin-resistant human oral cancer.

Chitosan, derived from chitin, is the only natural basic cationic polymer with good biodegradability, biocompatibility, cytophilicity, and hemocompatibility [21,22]. Moreover, it is commonly used in functional biomaterials. However, its poor water solubility limits its further application. Fortunately, chitosan has a unique parental backbone structure with abundant active hydroxyl and amino groups in the molecular chain, which can be chemically modified [23,24]. For the first time, we synthesized a new amphiphilic chitosan (CS—SA—DA), which is safe, non-toxic, and has a high drug-carrying capacity, by grafting succinic anhydride (SA) and deoxycholic acid (DA) onto chitosan amino groups. Amphiphilic chitosan has both hydrophilic and hydrophobic groups, and it can self-assemble to form super-stable micelles when dissolved in water [25,26]. N-succinyl chitosan, synthesized by the reaction of succinic anhydride with chitosan, has good biocompatibility and low toxicity, and it is commonly used in drug carriers and wound dressings. Deoxycholic acid is highly lipid-soluble and enhances the stability of the connection between the target drug and the wall. The compound can effectively interact with the apical-dependent bile acid transporter protein in the small intestine to enhance drug absorption [27]. It is often used as a hydrophobic side chain for amphiphilic polymers. In this study, the structure of the CS—SA—DA polymer was investigated by ^1^H-NMR spectroscopy, infrared spectroscopy (FTIR), and X-ray diffraction (XRD). Curcumin nanomicelles (ACS Cur) were prepared by encapsulating curcumin via an ultrasonic self-assembly method. The microscopic morphology, particle size, in vitro release behavior, safety, antioxidant activities, and anti-inflammatory activities of ACS Cur nanomicelles were investigated. The results indicate that nanomicelles can improve the water solubility, stability, antioxidant activities, and anti-inflammatory activities of curcumin, achieve slow drug release, and reduce the dosing frequency.

## 2. Results and Discussion

### 2.1. Characterization of Amphiphilic Chitosan

**Figure 1:**
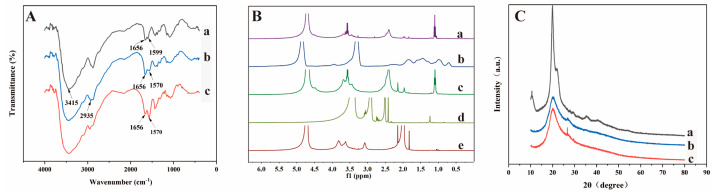
(**A**) Infrared spectra of CS (a), CS—SA (b), and CS—SA—DA (c). (**B**) Nuclear magnetic resonance hydrogen spectra of CS—SA—DA (a), DA (b), CS—SA (c), SA (d), and CS (e). (**C**) X-ray diffractograms of CS (a), CS—SA (b), and CS—SA—DA (c).

The acylation reaction of succinic anhydride and deoxycholic acid with chitosan C_2_-NH_2_ resulted in amphiphilic chitosan (CS—SA—DA). The results of its FTIR spectrum analysis are shown in Figure 1A(c). The basic characteristic absorption bands of chitosan were detected at 3415 cm^−1^ (primary amine stretching) and 1599 cm^−1^ (N-H bending vibration in amide). The relatively strong absorption signal at 1656 cm^−1^ represented the C=O stretching vibration in amides. The absorption bands of both amide signals were enhanced and slightly shifted toward a lower wavenumber compared to the chitosan IR spectrum (Figure 1A(e)), indicating that the reaction occurred to form amide groups on the primary amine sites of chitosan. In addition, the spectrum showed the same enhancement of the absorption bands at 2935 cm^−1^, which represented the vibrations of methylene, indicating the successful grafting of succinic anhydride [28,29]. In Figure 1A(c), the absorption band at 1570 cm^−1^ is significantly enhanced compared to Figure 1A(a), again indicating that the reaction occurred at C_2_-NH_2_, at which the amide group was formed [30]. In conclusion, amphiphilic chitosan was successfully synthesized.

In addition, the structures of chitosan and its derivatives were characterized by ^1^H-NMR. The ^1^H-NMR spectra of CS, SA, DA, CS—SA, and CS—SA—DA are shown in Figure 1B. The proton signal of chitosan in CS—SA—DA was shown in the 3-4ppm of 1H NMR spectra (Figure S1). A new absorption peak was detected at 2.25–2.5 ppm, which was the methylene proton signal of succinic anhydride at a higher field [31]. In Figure 1B(a), compared with the spectrum of Figure 1B(c), a new absorption peak also appears at 0.68–1.12 ppm, which is the methyl proton signal of deoxycholic acid [32]. In addition, the grafting rate of SA was calculated to be 46.94% by NMR hydrogen spectroscopy.

The X-ray diffraction patterns of CS, CS—SA, and CS—SA—DA are shown in Figure 1C. The strong diffraction peak at 15°–25° in Figure 1C(a) can be attributed to the free amino group and the hydrogen bonding in the two-dimensional crystal structure of chitosan. In Figure 1C(b), the XRD pattern was less intense, the peak area was wider, and the intensity of the characteristic peak was lower. This indicates that the grafting of SA onto chitosan amino groups reduced the number of hydrogen bonds within and between molecules while the natural crystalline structure of chitosan was deteriorating [17]. Figure 1C(c) shows a similar behavior of the X-ray diffraction pattern as in Figure 1C(b) due to the grafting of DA onto chitosan, which decreased the crystallinity. In addition, a new peak appeared at 2θ = 27° for CS—SA—DA, which may be attributable to the change in the polycrystalline structure from the non-thermodynamically stable initial crystalline form to the most stable form due to the addition of DA to CS—SA. [33]. This is in agreement with the results of Lin Yue et al. [34]. The above results indicate the successful synthesis of CS—SA—DA.

The results of the elemental analysis are shown in Table 1. The grafting rate of DA was calculated as 6% based on the results.

### 2.2. Critical Micelle Concentration of Blank Nanomicelles

**Figure 2:**
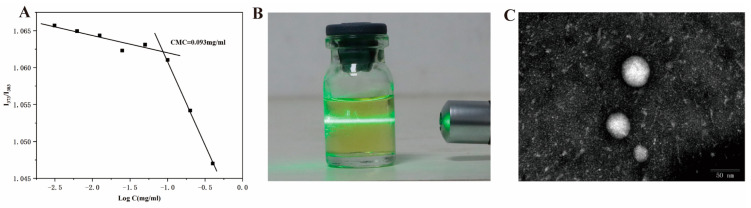
(**A**) Critical micelle concentration of ACS. (**B**) Tindal effect map of ACS Cur. (**C**) TEM image of ACS Cur (staining with 0.5% phosphotungstic acid; scale bar = 50 nm).

Amphiphilic chitosan contains both hydrophilic and hydrophobic chains and can self-assemble in aqueous solution to form nanocarriers with a core–shell structure, a behavior triggered by hydrophilic–hydrophobic interactions between the polymer chains [35]. The concentration of amphiphilic chitosan solution needs to be greater than or equal to its critical micelle concentration for the polymer to self-assemble and aggregate to form polymeric micelles with a core–shell structure. Therefore, determining the critical micelle concentration is crucial for preparing ACS Cur. Pyrene is very sensitive to the polarity of the microenvironment, and the intensity ratio of the first peak (I_1_, 373 nm) to the third peak (I_3_, 383 nm) in its fluorescence spectrum is always used to describe this subtle variation [36]. The results of the critical micelle concentration of blank nanomicelles are shown in Figure 2A. The critical micelle concentration of the blank nanomicelles was 0.093 mg∙mL^−1^, indicating that CS—SA—DA can self-assemble to form nanomicelles at lower concentrations. Figure 2A shows that the fluorescence intensity ratio (I_373_/I_383_) at 373 nm and 383 nm decreased slowly and then rapidly as the concentration of blank nanomicelles increased. When the concentration of amphiphilic chitosan is lower than CMC, ACS exists in the form of molecular chains in water, while pyrene molecules dissolve in water. When the concentration of amphiphilic chitosan is higher than that of CMC, it will self-assemble to form micelles with shell and nucleus structures. The fluorescent probe pyrene will be embedded in the nanomicelles. Pyrene will be transferred from the polar environment to the hydrophobic microregion of the nonpolar nanomicelles, which leads to the mutation of I_373_/I_383_ values.

### 2.3. Particle Size, Zeta Potential, and Micromorphological Analysis of ACS Cur

The average particle size of blank nanomicelles and ACS Cur was determined by nanoparticle size potentiometry (Table 2). The particle size of ACS Cur was significantly larger than that of the blank nanomicelles, probably due to the encapsulation of curcumin, which increased the particle size. The PDI values of ACS and ACS Cur were both lower than 0.7, indicating that their particle size distribution range is smaller and more stable [37]. Both blank nanomicelles and ACS Cur were negatively charged and stable. Zeta potential is often used to indicate the stability of a particle system. When the zeta potential of nanoparticles is high, the particles can repel each other, preventing aggregation and, thus, increasing the stability of the solution. The high value of the zeta potential of ACS indicates that the system is stable. After adding Cur to ACS, the zeta potential decreased from −47.28 ± 7.21 mV to −20.33 ± 0.25 mV, which is in agreement with the observation previously reported by Yuting Yuan et al. [38]. The Tindal effect is a classical method of qualitatively distinguishing between solutions and micellar suspensions [39]. A “bright pathway” in the micelles can be observed when the light beam passes through the micelles. Figure 2B shows that ACS Cur is yellow and uniformly colored at room temperature. When illuminated with a laser, the ACS Cur suspension shows a bright “pathway”, indicating the presence of nanomicelles. Figure 2C shows the transmission electron microscope image of ACS Cur, which shows that ACS Cur has a spherical morphology, showing the vesicle structure. The particle size of ACS Cur measured by DLS and TEM was different, mainly because the particle size of DLS was measured in aqueous solution, while the TEM image was obtained in a dry state. These phenomena are similar to those reported in the literature [40,41].

### 2.4. Encapsulation Rate (EE) and Drug Loading Rate (DL) of ACS Cur with In Vitro Release

**Figure 3:**
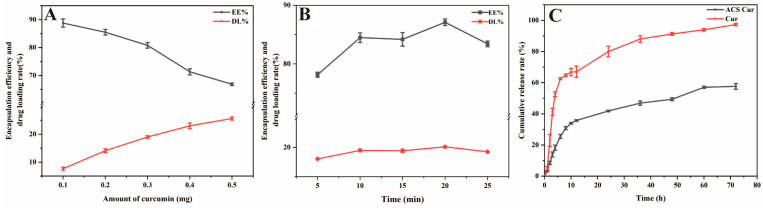
(**A**) Effects of different curcumin inputs on the encapsulation and loading rates. (**B**) Effects of different sonication times on encapsulation rate and drug loading rate. (**C**) In vitro release profiles of free Cur and ACS Cur.

The amount of curcumin was determined by UV–visible spectrophotometry, and the encapsulation rate and drug loading rate of ACS Cur were calculated by linearly fitting the standard formula. Figure 3A shows the effects of different curcumin inputs on the encapsulation rate and drug loading rate of ACS Cur. When the curcumin input increased from 0.1 mg to 0.5 mg, the encapsulation rate decreased from 88.80 ± 1.47% to 66.84 ± 0.46%. This significant decrease in encapsulation efficiency may be due to the fact that the dosage of Cur exceeded the loading capacity of the nanomicelles. This is consistent with the results of Joung-Pyo Nam et al. [42]. In addition, the loading rate increased from 7.64 ± 0.53% to 25.62 ± 0.52%. The higher encapsulation rate and drug loading rate of 80.80 ± 0.99% and 19.02 ± 0.46%, respectively, were obtained when the curcumin input was 0.3 mg, so the curcumin input was selected as 0.3 mg. Figure 3B shows the effect of sonication time on the encapsulation rate and drug loading rate of ACS Cur. The results show that the encapsulation and drug loading rate decreased after the first increase in sonication time. When the sonication time was increased from 5 min to 20 min, the encapsulation rate increased from 78.15 ± 0.42% to 87.11 ± 0.56%, and the drug loading rate increased from 18.03 ± 0.097% to 20.10 ± 0.13%. The results show that the best encapsulation and drug loading rates were demonstrated at 20 min of sonication time. After that, the sonication procedure may affect the ACS Cur, disrupting the micelles. The core material was released, leading to a gradual decrease in the encapsulation and drug loading rates. The above results show that ACS has a good encapsulation effect on curcumin and can significantly improve the water solubility of curcumin.

The release of drug-laden nanomicelles in the blood can be simulated in PBS solution at pH 7.4 [43]. The in vitro release profile of ACS Cur is shown in Figure 3C. The results indicate that the release rate of ACS Cur is significantly slower than that of free Cur, and the cumulative release rate only reaches 57.63 ± 1.81% at 72 h of release, showing a slow release effect. Before 8 h, ACS Cur was in the abrupt release phase with an explosive release, followed by a slower release rate, and then finally entered the stable phase, showing a slow release. The above results indicate that the prepared ACS Cur can achieve a slow release effect, prolong the therapeutic time of curcumin in vivo, and reduce the number of doses necessary.

### 2.5. Stability and Antioxidant Capacity of ACS Cur

**Figure 4:**
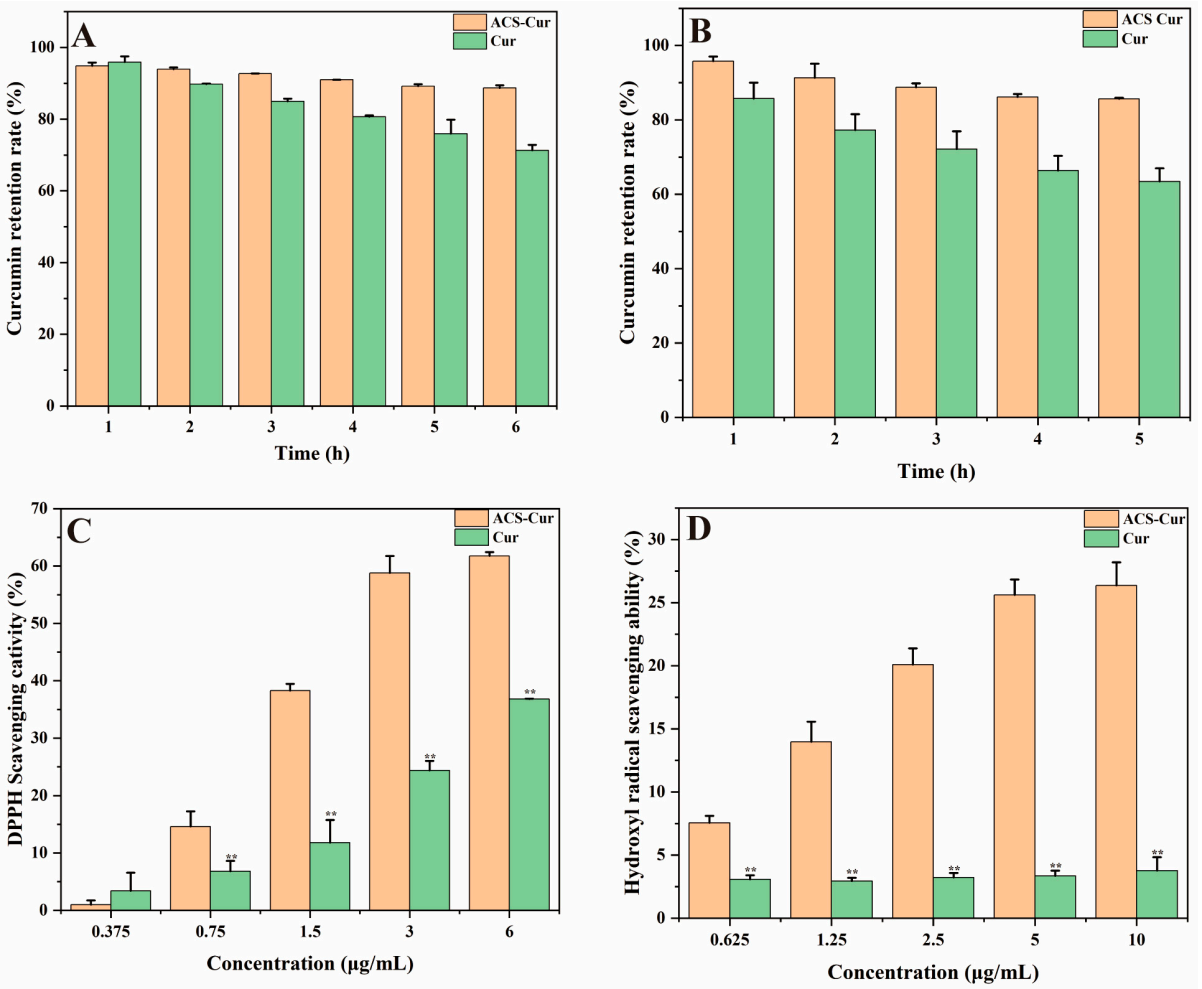
(**A**) Effect of temperature (70 °C) on ACS Cur. (**B**) Effect of pH (8.0) on the stability of ACS Cur. (**C**) Ability of ACS Cur to scavenge DPPH radicals. (**D**) Ability of ACS Cur to scavenge hydroxyl radicals. Note: ** *p* < 0.01; significant difference compared with the same concentration of the ACS Cur group.

Curcumin is poorly soluble in water but soluble in a neutral–alkaline environment and decomposes rapidly [44]. The results in Figure 4A show that both ACS Cur and free Cur gradually degraded with increasing time at 70 °C, but free Cur decomposed faster, with 88.73 ± 0.73% retention of ACS Cur and 71.26 ± 1.58% retention of free Cur at 6 h. Similarly, the results in Figure 4B show that, at pH 8.0, both ACS Cur and free Cur gradually degraded with time, but free Cur decomposed faster, with 85.64 ± 0.31% retention of ACS Cur and 63.43 ± 3.54% retention of free Cur at 5 h. The decomposition rate of free Cur was significantly greater than that of ACS Cur (*p* < 0.05). In conclusion, ACS Cur can improve the stability of curcumin under high temperatures and alkaline conditions.

DPPH can react with any compound that releases hydrogen atoms or electrons, changing its color from purple to yellow. The degree of discoloration is quantitatively related to the number of electrons, making it available for rapid quantitative free radical scavenging [45]. The scavenging ability of both ACS Cur and free Cur for DPPH radicals increased with increasing curcumin concentration (Figure 4C). The scavenging ability of ACS Cur for DPPH radicals was significantly higher than that of free Cur at concentrations ranging from 0.75 to 6 μg∙mL^−1^ (*p* < 0.05). Hydroxyl radicals are the most powerful oxidative radicals, which react with most biological macromolecules in the body, causing damage [46]. The scavenging ability of ACS Cur for hydroxyl radicals was significantly higher than that of free Cur (*p* < 0.05) at 0.375–6 μg∙mL^−1^ (Figure 4D). In conclusion, ACS Cur improves the antioxidant capacity of curcumin.

### 2.6. ACS Cur Hemocompatibility and Safety Assessment

**Figure 5:**
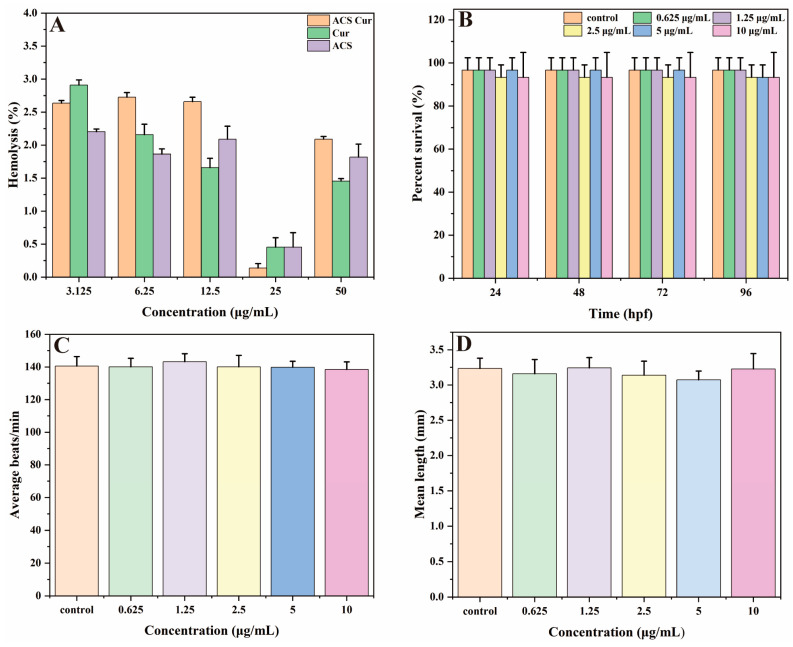
(**A**) Hemocompatibility of ACS Cur, free Cur, and ACS. (**B**) Effect of ACS Cur on the survival rate of zebrafish. (**C**) Effect of ACS Cur on the heart rate of zebrafish. (**D**) Effect of ACS Cur on the body length of zebrafish.

Most studies have shown that the interaction of free amino groups of chitosan with plasma proteins or blood cells can induce thrombosis or hemolytic reactions [47,48]. Studies have shown that polymers can enter circulation directly or indirectly, actively or passively, and rapidly or slowly. Their contact with the blood can be harmful or even instantly fatal [48], so it is important to determine the hemocompatibility of materials. Hemolysis is considered to be a very simple and reliable measurement to assess the hemocompatibility of materials [47]. Different concentrations of ACS Cur, free Cur, and ACS were incubated with erythrocytes to observe their effects on erythrocytes. The results in Figure 5A show that the hemolysis rates of different concentrations of ACS Cur, free Cur, and ACS were well below the international standard value (5%) [27], indicating that all samples had good hemocompatibility.

Zebrafish are widely used in toxicological and immunological studies due to their small size, ease of reproduction, high spawning rate, transparent embryos, and highly similar genome and immune system to humans [49]. In the present study, zebrafish embryos were exposed to different concentrations of ACS Cur (0, 0.625, 1.25, 2.5, 5, and 10 μg∙mL^−1^) for 96 h to study the developmental toxicity of ACS Cur on zebrafish embryos. The results showed that the survival rate, heart rate, and body length of the groups treated with ACS Cur were not significantly different from those of the blank group. The high survival rate of zebrafish in each group indicated that ACS Cur was not toxic to the development of zebrafish embryos in this concentration range.

### 2.7. The Anti-Inflammatory Capacity of ACS Cur

**Figure 6:**
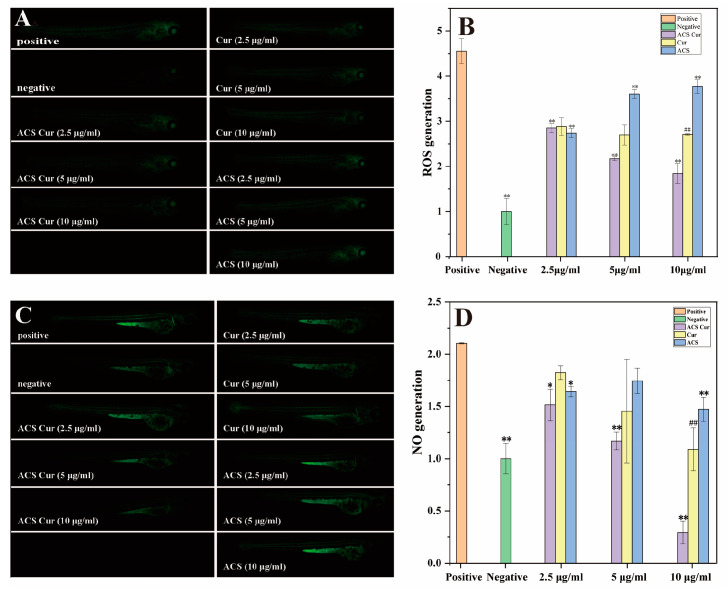
(**A**,**B**) Effects of ACS Cur, free Cur, and ACS on ROS production in zebrafish embryos induced by LPS. (**C**,**D**) Effects of ACS Cur, free Cur, and ACS on NO production in zebrafish embryos induced by LPS. Note: * means a significant difference compared with the positive group (*p* < 0.05); ** means a significant difference compared with the positive group (*p* < 0.01), and ## means a significant difference compared with the same concentration of the Cur group (*p* < 0.01).

Curcumin has a well-known good anti-inflammatory effect. To evaluate it [10], embryonic zebrafish inflammation was induced by LPS, and drug intervention was given to detect the production of ROS, NO, and related inflammatory factors. DCF-DA and DAF-FMDA fluorescent dyes were used to detect the production of ROS and NO in the zebrafish. The results (Figure 6A,B) show that the LPS group (positive group) produced the highest ROS content in zebrafish. The difference was significant compared with the blank control group (negative group) (*p* < 0.01), indicating that LPS can induce an inflammatory response and oxidative stress in zebrafish embryos. Compared with the LPS group, different concentrations of ACS Cur, Cur, and ACS groups could all inhibit the production of ROS (*p* < 0.01), indicating that they could all inhibit LPS-induced oxidative stress. The inhibition of ROS in the ACS Cur group was concentration-dependent. In particular, ACS Cur at a 10 μg∙mL^−1^ concentration inhibited ROS generation significantly more than Cur at the same concentration (*p* < 0.01). Figure 6C,D show the NO production. The results show that, compared with the LPS group, the fluorescence intensity of the group without LPS was also significantly weakened, indicating that LPS can induce inflammatory reactions in zebrafish while it promotes NO production. Compared with the LPS group, both the ACS Cur and Cur groups could inhibit NO production (*p* < 0.01), and the inhibition of NO by the ACS Cur group also showed concentration-dependency. In particular, ACS Cur at a 10 μg/mL concentration inhibited ROS generation significantly more than Cur at 10 μg/mL (*p* < 0.01). Through these results, it was concluded that ACS Cur could enhance the anti-inflammatory activity of ACS.

**Figure 7:**
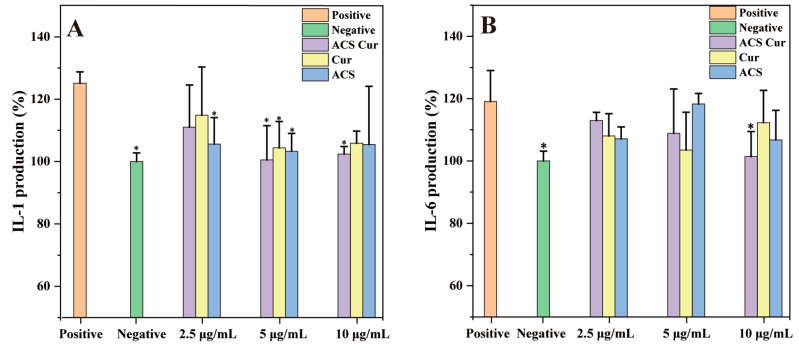
Effects of ACS Cur, Cur, and ACS on producing pro-inflammatory factors in LPS-stimulated zebrafish embryos: (**A**) production of IL-1; (**B**) production of IL-6; * means a significant difference compared with the positive group (*p* < 0.05).

Zebrafish embryos at 24 hpf were co-treated with different concentrations of the samples and LPS up to 72 hpf. Then, IL-1 and IL-6 were measured in the embryos by ELISA at 450 nm on an enzyme marker (Figure 7). The results showed that the production of IL-1 and IL-6 in zebrafish embryos increased significantly (*p* < 0.05) after the inflammatory response was induced with LPS. The levels of IL-1 and IL-6 in zebrafish treated with 10 μg∙mL^−1^ ACS Cur decreased significantly (*p* < 0.05), indicating that ACS Cur can inhibit the development of inflammation (by suppressing the release of IL-1 and IL-6). The production of IL-1 and IL-6 was less in the 10 μg∙mL^−1^ ACS Cur group compared to the 10 μg/mL Cur group, but the difference between the two groups was not statistically significant (*p* > 0.05).

## 3. Materials and Methods

### 3.1. Materials, Instruments, and Equipment

Chitosan (deacetylation ≥ 95%, molecular weight 100 KDa) was purchased from Kool Bioengineering Co., Ltd. (Hefei, China). Sodium hydroxide was obtained from Xilong Chemical Co., (Guangzhou, China). Deoxycholic acid (DA), succinic anhydride (SA), 1-ethyl-(3-dimethylamino-propyl) carbodiimide hydrochloride (EDAC), N-hydroxysuccinimide (NHS), curcumin (Cur, content ≥ 95%), pyrene, Tween 80, 1,1-diphenyl-2-trinitro phenylhydrazine (DPPH), 2,7-dichlorofluorescein diacetate (DCF-DA), and diaminofluorophore-4-amino-5-methylamino-2′7′-difluorofluorescein diacetate (DAF-FMDA) were bought from Maclean Biochemical Technology Co., Ltd. (Shanghai, China). Lipopolysaccharides were purchased from Solebro Biotechnology Co., (Seoul, Republic of Korea). Hydroxyl radical test kits were obtained from Nanjing Jiancheng Institute of Biological Engineering, Nanjing, China. Zebrafish interleukin 1 (IL-1) ELISA kits and zebrafish interleukin 6 (IL-6) ELISA kits were purchased from Jiangsu Enzyme Immunity Industry Co., (Yancheng, China). In addition, methanol, ammonia, glacial acetic acid, ethanol, acetone, and dimethyl sulfoxide (DMSO) were purchased from Guanghua Technology Co., (Shantou, China). All chemicals and reagents were of analytical grade and did not require further purification before use. Ultrapure water was used for all experiments.

The following instruments were used: IRAffinity-1S Fourier infrared spectrometer (Shimadzu, Kyoto, Japan); Smart Lab 9 kW X-ray diffractometer (Rigaku, Tokyo, Japan); AVANCE NEO 400 MHz NMR (Bruker, Karlsruhe, Germany); Elementar vario EL cube organic elemental analyzer (Elementar, Langenselbold, Germany); Malvern Zetasizer Nano ZS90 Nano Particle Size Potentiometer (Malvern Instruments Ltd., Malvern, UK); UV752N UV–Visible Spectrophotometer (Shanghai Precision Scientific Instruments Co. Corporation, Shanghai, China).

### 3.2. Synthesis of Chitosan Modified with Succinic Anhydride (SA)

An already available method was used [31] and modified. First, 2 g of chitosan was added to 40 mL of DMSO, stirred well, and then 4 g of succinic anhydride was slowly added and reacted at 65 °C for 6 h. The mixture was filtrated, and the precipitate was dissolved in 100 mL of ethanol at room temperature. The mixture was homogenized for 1 h. The pH was adjusted to 10–12 by a 1 mol·L^−1^ NaOH solution and then filtered. The product was dissolved in 90 mL of distilled water, and 270 mL of acetone was added. The end product was washed with ethanol and acetone three times (300 mL/time). Finally, the precipitate was dried under vacuum to obtain CS—SA.

### 3.3. Synthesis of Amphiphilic Chitosan (CS—SA—DA)

**Scheme 1:**
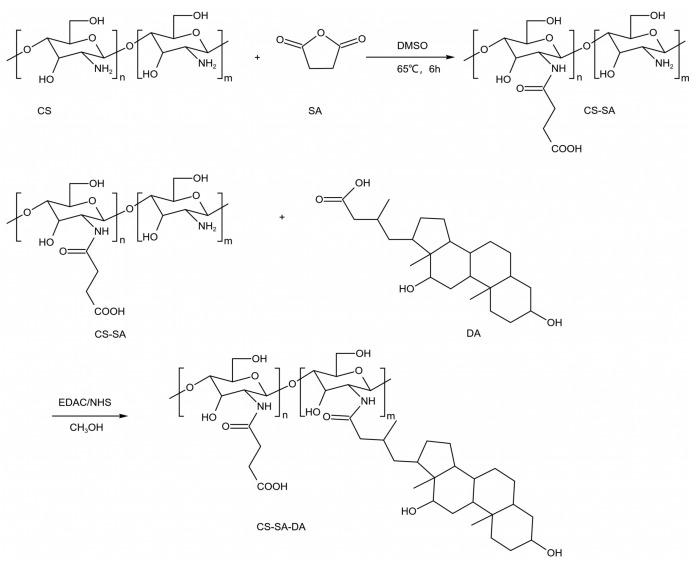
Synthesis route of CS—SA—DA.

Based on the literature [50], 200 mg of N-succinyl chitosan was dissolved in 10 mL of distilled water (solution A). DA (120 mg), EDAC (72 mg), and NHS (72 mg) were stirred in 10 mL of methanol for 1.5 h (solution B). Solution B was added dropwise to solution A and stirred at room temperature for 24 h. After that, the reaction mixture was added to 10 mL of methanol. The filtrate was transferred to 20 mL of ethanol/ammonia mixture (ethanol/ammonia = 17:3), filtered, and the precipitate was washed three times with anhydrous ethanol and methanol, separately, and dried under vacuum to obtain amphiphilic chitosan (CS—SA—DA). The synthesis route of CS—SA—DA is shown in Scheme 1.

### 3.4. Characterization of Amphiphilic Chitosan

The Fourier-transform infrared spectra of chitosan and its derivatives were determined using the potassium bromide compression method with a Fourier-transform infrared spectrometer. The ^1^H nuclear magnetic resonance spectra were recorded at 400 MHz. Chitosan was dissolved in 1% C_2_D_4_O_2_. Succinic anhydride and deoxycholic acid were separately dissolved in CD_4_O. Finally, CS—SA and CS—SA—DA were dissolved in D_2_O. Tetramethylsilane was the internal standard substance. The compounds were ultrasonicated for 10 min before testing. The grafting rate of SA was calculated by ^1^H NMR [51]. The 1H NMR spectrum of CS—SA—DA should show the proton signal of chitosan at 3–4 ppm and the methylene proton signal of succinic anhydride at a higher field of 2.25–2.5 ppm. In addition, the methyl proton signal of deoxycholic acid should appear at a high field of 0.68–1.12 ppm. The crystallization behavior of chitosan and its derivatives was analyzed using a Rigaku Smart Lab 9 kW X-ray diffractometer in the 10°–80° (2θ°) range. The C, H, and N contents in CS—SA and CS—SA—DA were determined by the Dumas combustion method using the Elementar vario EL cube organic elemental analyzer. The grafting efficiency of DA was calculated [52] according to Formula (1):

(1) $$\mathrm{DA}\%=\;\frac{{C/N}_{CS-SA-DA}-{C/N}_{CS-SA}}{24}\times 100\%$$

where C/N is the molar ratio of C and N atoms, while 24 is the number of carbon atoms in deoxycholic acid.

### 3.5. Determination of the Critical Micelle Concentration (CMC) of Blank Nanomicelles (ACS)

The critical micelle concentration of the blank nanomicelles was determined using pyrene as a molecular probe [53]. First, 40 mg of CS—SA—DA was dissolved in 10 mL of distilled water and sonicated for 10 min at room temperature to obtain a blank nanomicelles master batch (ACS) at a concentration of 4 mg∙mL^−1^, which was diluted to 0.4, 0.2, 0.1, 0.05, 0.025, 0.0125, 0.00625, and 0.003125 mg∙mL^−1^. A concentration of 50 μL of 6∙10^−5^ mg∙mL^−1^ pyrene/acetone solution was added to 5 mL of the above different concentration dilutions, mixed, and shaken well. Then the mixture was sonicated for 15 min, heated at 50 °C for 2 h, kept at room temperature, and protected from light overnight to reach equilibrium. The fluorescence intensity of the blank nanomicelles (373 nm and 383 nm sized) was measured using a multifunctional microplate detection system. The excitation wavelength was set to 335 nm. The absorption peak intensity ratio was used the logarithm of the concentration as the horizontal coordinate, and the I_373_/I_383_ ratio as the vertical coordinate.

### 3.6. Preparation of ACS Cur

ACS Cur was prepared by an ultrasonic self-assembly method. First, 1 mg of amphiphilic chitosan powder was weighed, dissolved in 10 mL of ultrapure water, and sonicated (120 W, 40 kHz) for 10 min at room temperature. Then, 10 mg of curcumin was weighed and dissolved in 3 mL of anhydrous ethanol, and a certain amount of curcumin solution was slowly added dropwise to the blank nanomicelles. The mixture was shaken for 20 min, and then the solvent was evaporated using a hot-water bath at 65 °C. The evaporation continued until the solution became turbid. The mixture was sonicated for 15 min at room temperature to obtain the ACS Cur, which was centrifuged (4000 rpm/min, 10 min). The supernatant was ACS Cur, and the precipitate was the unembedded curcumin.

### 3.7. Particle Size, Zeta Potential, and Morphological Characterization of ACS Cur

The particle size and zeta potential of ACS and ACS Cur were evaluated by nanoparticle size potentiometry. The Tyndall effect verified the formation of ACS Cur [54], and TEM was used to observe the microscopic morphology of ACS Cur. Before observing the morphology by TEM, 10 μL of the sample was added dropwise onto a copper grid for 1 min, and the floating liquid was removed by filter paper. We then added 10 μL of phosphotungstic acid staining solution onto the copper mesh for 1 min, and then we removed the floating liquid with filter paper again. The sample was dried at room temperature for a few minutes before observation and image acquisition under a transmission electron microscope.

### 3.8. Measurement of Encapsulation Rate and Drug Loading Rate of ACS Cur

After centrifugation of the ACS Cur mixture, the precipitate was re-dissolved with 10 mL of anhydrous ethanol and then centrifuged again (4000 rpm/min, 10 min). The supernatant was diluted with anhydrous ethanol, the absorbance was measured at 425 nm using a UV spectrophotometer, and the unencapsulated curcumin content was calculated by a linear regression formula (Y = 146.51X + 0.0009, r^2^ = 0.9994). The encapsulation and drug loading rates were calculated according to Formulae (2) and (3):

EE = (amount of encapsulated curcumin quality)/(total curcumin mass) × 100%(2)

DL = (amount of encapsulated turmeric mass)/(total mass of curcumin + mass of amphiphilic chitosan) × 100%(3)

### 3.9. ACS Cur In Vitro Sustained Release Performance

The dialysis method was used to study the ACS Cur release. Referring to the method in [55], with modifications, 10 mL of ACS Cur and free Cur solution was added to dialysis bags, with a cut-off molecular weight of 3500 D. The bags were immersed in 45 mL of PBS buffer (containing 0.5% Tween 80, pH = 7.4) and shaken at 37 °C. At specific time intervals, 4 mL of release medium was collected and replaced with 4 mL of fresh medium. Cur’s absorbance was measured using a UV spectrophotometer at 425 nm, and the cumulative release rate was calculated according to Formula (4):

(4) $$\mathrm{Cumulative}\;\mathrm{release}\;\mathrm{rate}=\frac{\sum_{i=2}^{n-1}\left(4\times C_{i-1}\right)+45\times C_{n}}{C_{0}V_{0}}\;\times \;100\%$$

where $C_{n-1}$ is the drug concentration in the *n*-1st release medium, $C_{n}$ is the drug concentration in the nth release medium, *C*_0_ is the drug concentration in the original ACS Cur, and *V*_0_ is the volume of the nanomicelles in the dialysis bag.

### 3.10. The Stability of ACS Cur

Free Cur and ACS Cur solutions with equivalent concentrations of curcumin were prepared and diluted by half with a pH 8.0 phosphate buffer. The absorbance values were measured after 0, 1, 2, 3, 4, and 5 h. The changes in curcumin content at different times were calculated by considering the drug content at 0 h as 100%. The thermal stability of ACS Cur was determined as described in [55]. Free Cur and ACS Cur solutions were incubated at 70 °C. The remaining Cur content was determined by measuring the maximum absorbance at 425 nm wavelength on an enzymatic calibrator at predetermined time intervals.

### 3.11. Antioxidant Activity of ACS Cur

The antioxidant activity of the nanomicelles was evaluated using the DPPH radical scavenger, a stable purple radical that can acquire an electron from the antioxidant. The method was modified as described in a previous study [56]. Specifically, free Cur and ACS Cur solutions were prepared at concentrations of 0.375, 0.75, 1.5, 3, and 6 μg∙mL^−1^. DPPH ethanol stock solution was prepared at a concentration of 1 mM and stored in the dark, and 2 mL of free Cur and ACS Cur solutions at different concentrations was added to 2 mL of DPPH ethanol solution at a concentration of 0.1 mM, shaken, and immediately stored in the dark for 45 min. The absorbance at 517 nm was measured by a UV spectrophotometer, with ultrapure water as a blank control, using Formula (5) to calculate the DPPH radical scavenging rate:

Scavenging activity (%) = (A_0_ − A_S_)/A_0_ × 100 (5)

where A_0_ is the absorbance of the DPPH ethanol solution and ultrapure water, and A_S_ is the absorbance of the DPPH ethanol solution and sample.

The hydroxyl radical scavenging ability of free Cur and ACS Cur solutions was measured using a hydroxyl radical test kit.

### 3.12. Hemocompatibility of ACS Cur

The toxicity of the nanomicelles was tested using the release of hemoglobin from erythrocytes according to a previously described colorimetric method [57]. Briefly, blood was collected from the hearts of six SD rats into centrifuge tubes containing heparin, centrifuged at 3000 rpm for 10 min, and then washed three times with saline. The purified RBCs were resuspended in saline to obtain a 2% (*v*/*v*) suspension of RBCs. Then, 2.0 mL of RBC suspension was added to 2.0 mL of different concentrations of ACS, free Cur, and ACS Cur. After incubation at 37 °C for 1 h, the mixture was centrifuged at 3000 rpm for 15 min. The supernatant was collected, and the absorbance was measured at 540 nm. Normal saline solution was used as the negative control for 0% hemolysis, while ultrapure water was used as a positive control for 100% hemolysis. The percentage of hemolysis was calculated according to Formula (6):

(6) $$\mathrm{Hemolysis}\;(\%)=\frac{A_{Sample}-A_{Saline}}{A_{Water}-A_{Saline}}\times 100\%$$

### 3.13. Safety Assessment of ACS Cur

The method in reference [49] was modified using wild-type (AB strain) zebrafish cultured in aerated water at 27 ± 1 °C. Light and dark cycling was set to 14 h of light and 10 h of darkness. The fish were fed with pellets in the morning and hatchery fenugreek shrimp at noon and evening. The night before spawning, male and female fish were placed in the mating tank at a 1:1 ratio, with a partition separating the males and females. The embryos were randomly transferred to 24-well plates, with ten embryos per well, and exposed to different concentrations of ACS Cur solutions (0, 0.625, 1.25, 2.5, 5, and 10 μg∙mL^−1^) for 96 h. Each concentration was repeated three times and changed every 24 h. Morphological changes in the zebrafish embryos were observed at 24, 48, 72, and 96 hpf, and survival rates were calculated using a stereomicroscope. Body length and heart rate were measured at 96 hpf.

### 3.14. Anti-Inflammatory Activity of ACS Cur

NO production in zebrafish was measured using the fluorescent probe dye DAF-FMDA, and ROS production in zebrafish was detected using the fluorescent probe dye DCF-DA [58]. We collected 330 wild-type zebrafish at 9 hpf into 24-well plates, with 10/group, and three parallel groups were set up and pretreated with different concentrations of drugs for 1 h. An equal volume of 10 μg/mL LPS was added to the well plates of the drug administration and model groups, and an equal volume of aeration water was added to the blank group. After incubation at 28 °C for 24 h, the zebrafish embryos of each group were washed with culture water, incubated at a constant temperature until 72 hpf, and treated with aeration water containing 10 μM DAF-FMDA or 20 μg∙mL^−1^ DCF-DA. They were incubated at 28 °C in the dark for 1 h for the ROS assay and 2 h for the NO assay. After incubation, they were washed with aeration water and anesthetized before observation. Images of stained embryos were observed using a spinning-disk super-resolution laser confocal microscope, and the relative amounts of ROS and NO in the zebrafish were measured using ImageJ software. ELISA kits were used to measure the contents of the inflammatory factors IL-1 and IL-6 in embryos.

### 3.15. Statistical Analysis

Origin 2021 software was used for statistical analysis; all data were expressed as the mean ± SD, and a one-way ANOVA test was used to determine statistical significance. Differences were significant at * *p* < 0.05 and ** *p* < 0.01.

## 4. Conclusions

In this study, novel amphiphilic chitosan was synthesized by grafting hydrophilic SA and hydrophobic DA, using chitosan as the raw material, followed by the preparation of ACS Cur by ultrasonic self-assembly, which is simple, inexpensive, and does not require the introduction of toxic organic solvents. The prepared blank nanomicelles had a low critical micelle concentration and good encapsulation ability for curcumin. ACS Cur undergoes a burst release early at 37 °C, after which the drug is slowly released, prolonging the half-life of curcumin. The maximum measured hemolysis rate of ACS Cur was 2.73 ± 0.068%, which meets the requirements of international standards. The amphiphilic chitosan showed improved aqueous solubility and hemocompatibility. The prepared ACS Cur improved the aqueous solubility, pH stability, thermal stability, in vitro antioxidant capacity, and in vivo anti-inflammatory capacity of curcumin. Therefore, this novel drug carrier may provide a new alternative for effectively delivering hydrophobic drugs like curcumin and a new reference for its expanded application.

## Figures and Tables

**Table 1:** Elemental analysis results of CS—SA and CS—SA—DA ($\bar{x\;}\;$± s, n = 3).

Samples	W (C)%	W (N)%	W (H)%	n (C)/n (N)
CS—SA	38.66 ± 0.43	4.41 ± 0.05	6.08 ± 0.33	10.23
CS—SA—DA	38.70 ± 0.12	3.87 ± 0.01	6.18 ± 0.07	11.67

**Table 2:** Particle size and zeta sites of blank nanomicelles and curcumin nanomicelles ($\bar{x}\;$± s, n = 3).

Sample	Diameter (nm)	PDI	Zeta (mV)
ACS	227.62 ± 17.14	0.28 ± 0.03	−44.32 ± 5.12
ACS Cur	269.36 ± 35.62	0.37 ± 0.07	−29.25 ± 0.32

## Data Availability

Data will be made available upon request from Chen Qizhou (sj13423409060@gmail.com) and Ouyang Qianqian (oyqq617@gdmu.edu.cn).

## Supplementary Materials

The following supporting information can be downloaded at: https://www.mdpi.com/article/10.3390/molecules29112693/s1, Figure S1: 1H-NMR spectra of CS—SA—DA (400 MHz).
