# Supplementary figures and images for: Preparation, Characterization, and Antioxidant Properties of Self-Assembled Nanomicelles of Curcumin-Loaded Amphiphilic Modified Chitosan
Source: Molecules. 2024 Jun 6;29(11):2693. doi: 10.3390/molecules29112693 (PMC11173681; doi:10.3390/molecules29112693)

## Figure captions

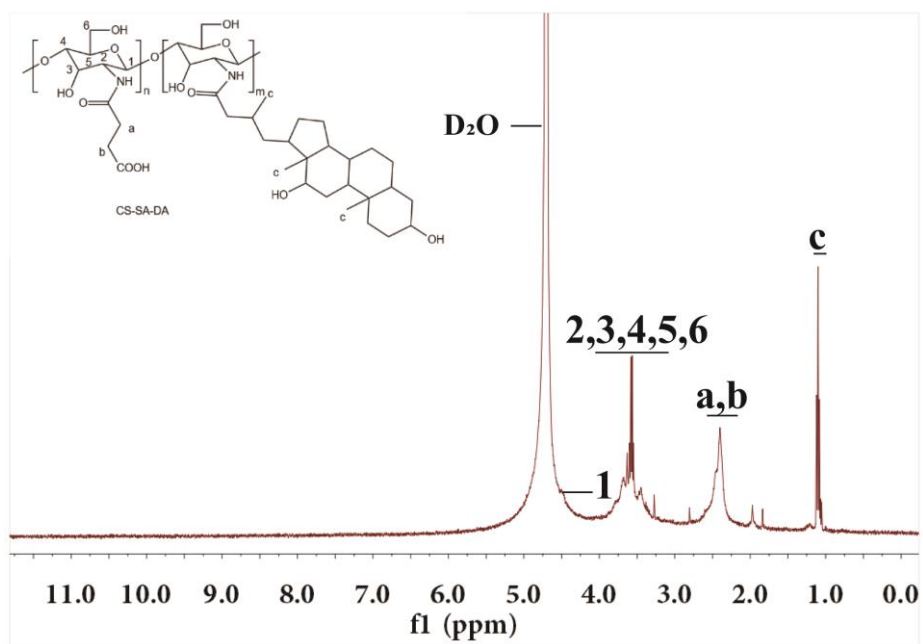

Fig. S1.  $^1\text{H}$ -NMR spectra of CS-SA-DA (400MHz).

Supplement: Supplementary file 1 [file molecules-29-02693-s001.zip › molecules-2971664-supplementary.pdf]
